# Active circulation of varicella zoster virus among different age groups in Sudan

**DOI:** 10.1017/S0950268822001923

**Published:** 2022-12-14

**Authors:** Omer Adam, Ahmed Musa, Amani Kamer, Safiya Benharrat, Judith M. Hübschen

**Affiliations:** 1 Department of Medical Biotechnology, Commission for Biotechnology and Genetic Engineering, National Centre for Research, Khartoum, Sudan; 2 Institute of Endemic Diseases, University of Khartoum, Khartoum, Sudan; 3 Department of Paediatrics, Faculty of Medicine, Alzaeim Alazhari University, Khartoum, Sudan; 4 Clinical and Applied Virology, Department of Infection and Immunity, Luxembourg Institute of Health, Esch-sur-Alzette, Luxembourg

**Keywords:** Chickenpox, varicella zoster, virology

## Abstract

In Sudan, data on varicella infections are lacking and the vaccine is currently not in use. The aim of this study was to investigate previous exposure to varicella zoster virus (VZV) among children and adults from the general population and among health-care workers (HCWs) in Khartoum. Dried blood spot samples collected between 2015 and 2016 from 294 children aged 1‒15 years, 153 adult volunteers and 241 HCWs were investigated for the presence of VZV IgG antibodies using ELISA. The overall seroprevalence of VZV IgG antibodies among the investigated cohorts was 50.4%, ranging between 14.3% in children and 79.3% in HCWs. Seropositivity increased with age among children and HCWs (*P* ⩽ 0.05). A relatively low seropositivity (64.7%) was observed among young adults and HCWs, suggesting that a high proportion of Sudanese adults remain susceptible. In hospital settings, this result implies a risk of nosocomial infection involving both HCWs and vulnerable patients. The results of this first VZV study in Sudan suggest active virus circulation in different age groups. Especially HCWs at the start of their career might benefit from vaccination, not only to save themselves from herpes zoster and its sequelae, but also to indirectly protect vulnerable patients.

Varicella (chicken pox) is caused by varicella zoster virus (VZV). It is one of the most common and contagious childhood infections and characterized by febrile vesicular rash. The disease can lead to serious complications or death especially in adults, infants and immunocompromised individuals. Varicella infection in pregnancy may lead to congenital defects (~1%), if acquired in the first two trimesters of pregnancy [1]. The defects include scarring on the skin, hypoplastic limbs and some eye and brain abnormalities [2]. Following primary infection, VZV can persist in peripheral autonomic ganglia and virus reactivation may result in herpes zoster (shingles) characterized by painful, localized vesicular rash [3]. Varicella is a vaccine preventable infection; however, the live attenuated vaccine is currently only used in about 25% of World Health Organization (WHO) member states [4]. In Sudan, no varicella vaccine is used, varicella infections are not notifiable and data on disease epidemiology or outbreaks are lacking. Therefore, the aim of this study was to investigate the extent of previous exposure to VZV among children and adults from the general population and among health-care workers (HCWs) in Khartoum, Sudan.

Dried blood spot (DBS) samples collected during a previous cross-sectional study on the seroprevalence of measles, mumps and rubella in Khartoum state, Sudan between 2015 and 2016 and stored at −80 °C were used [5]. Briefly, samples had been collected from 294 children aged 1‒15 years (mean 7.7 years) attending three major paediatric hospitals, 153 adult volunteers between 21 and 60 years (mean 36.2 years) working at the National Centre for Research and 241 HCWs aged 22‒61 years (mean 38.2 years) working at two major paediatric hospitals in Khartoum, Sudan. We used these samples to circumvent the challenges related to collecting, preserving and transporting new samples and because there has been no policy change related to varicella in Sudan since they had been collected. Sample integrity was assessed by re-testing 40 randomly selected sera for measles IgG antibodies using the Euroimmun anti-Measles ELISA (IgG) kit (EI 2610-9601 G, Lübeck, Germany) to verify that the results were qualitatively and quantitatively concordant with our previous findings. After we made sure that there were no issues with sample quality, we performed the extraction of serum from the DBS samples and the detection of VZV IgG antibodies as described in the Euroimmun anti-VZV ELISA (IgG) kit manual (EI 2650-9601 G, Lübeck, Germany). All samples with equivocal results were retested and categorized according to the second test result. Individuals with a second equivocal result were considered as seronegative during data analysis. Using the SPSS software, version 20 (SPSS Inc., Chicago, Illinois, USA), the categorical data were analysed by Pearson's *χ*^2^ test and *P*-values of <0.05 were considered statistically significant. The protocol of this study was submitted to the National Health Research Ethics Committee, Federal Ministry of Health, Sudan. The committee approved the testing of the stored DBS samples collected in the context of the previous study [5] for IgG antibodies against VZV. Informed consent was obtained from all participants for the initial study [5].

The overall seroprevalence of VZV IgG antibodies in Khartoum state was 50.4%, ranging between 14.3% in children, 74.5% in adult volunteers and 79.3% among HCWs (Table 1). As shown in Figure 1, a significant difference in seropositivity was observed between children and volunteer adults from the general population (*P* < 0.001). The seropositivity in children increased significantly with age from 6.0% among 1‒5 years old to 24.5% among 11‒15 years old (*P* = 0.001). Age was also correlated with seropositivity in adults, which ranged from about 65% among both volunteers and HCWs aged 21‒30 years to 78.7% (*P* = 0.143) and 86.6% (*P* = 0.009) among those aged ⩾41 years, respectively. In contrast, there was no significant difference between seropositivity of volunteer adults from the general population and HCWs (*P* = 0.272). Although not significant either, nurses showed a higher seropositivity (82.8%) compared to other occupational groups (*P* = 0.495). Neither sex nor residence was associated with VZV seropositivity (*P* > 0.05) in any of the study groups (Table 1). Among the 350 participating women, 317 (90.6%) were of childbearing age (21–49 years) and more than a quarter of them (*n* = 81/317, 25.6%) were seronegative.

**Fig. 1.**
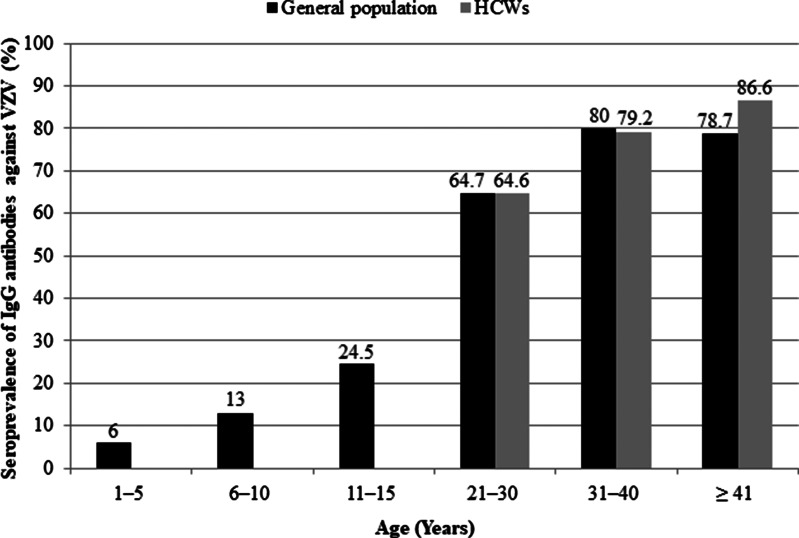
Seroprevalence of IgG antibodies against varicella zoster virus (VZV) by age group among children and adult volunteers from the general population and health-care workers (HCWs).

**Table 1.** Association between varicella zoster virus (VZV) antibody results and participant characteristics

Characteristic	IgG antibodies against VZV					
	Children		Adult volunteers		Health-care workers	
	Pos (%)	Neg (%)	Pos (%)	Neg (%)	Pos (%)	Neg (%)
Age (years)						
1‒5	6 (6.0)	94 (94.0)				
6‒10	13 (13.0)	87 (87.0)	–	–	–	–
11‒15	23 (24.5)	71 (75.5)				
21‒30			33 (64.7)	18 (35.3)	31 (64.6)	17 (35.4)
31‒40	–	–	44 (80.0)	11 (20.0)	76 (79.2)	20 (20.8)
⩾41^a^			37 (78.7)	10 (21.3)	84 (86.6)	13 (13.4)
	*P* = 0.001		*P* = 0.143		*P* = 0.009	
Sex						
Male	27 (17.3)	129 (82.7)	19 (76.0)	6 (24.0)	18 (94.7)	1 (5.3)
Female	15 (10.9)	123 (89.1)	95 (74.2)	33 (25.8)	173 (77.9)	49 (22.1)
	*P* = 0.115		*P* = 0.852		*P* = 0.083	
Region						
Bahri	8 (8.8)	83 (91.2)	38 (77.6)	11 (22.4)	84 (80.0)	21 (20.0)
Khartoum	14 (14.7)	81 (85.3)	37 (72.5)	14 (27.5)	20 (80.0)	5 (20.0)
Omdurman	20 (18.5)	88 (81.5)	39 (73.6)	14 (26.4)	87 (78.4)	24 (21.6)
	*P* = 0.147		*P* = 0.833		*P* = 0.953	
Job title						
Nurse	–	–	–	–	106 (82.8)	22 (17.2)
Physician					35 (72.9)	13 (27.1)
Lab technician					31 (77.5)	9 (22.5)
Vaccine worker					19 (76.0)	6 (24.0)
	–		–		*P* = 0.495	
Sub total	42 (14.3)	252 (85.7)	114 (74.5)	39 (25.5)	191 (79.3)	50 (20.7)
Total	294		153		241	

Pos, positive; Neg, negative.

a This age group was not further subdivided because of the limited number of participants and no significant seroprevalence increases.

While data on VZV exposure among African children are limited, our results are comparable to what was previously reported in Congo, where the overall seroprevalence of VZV antibodies was 8.0% among 7195 children and increased with age from 2% in 6 months old to 19% in 59 months old [6]. Moreover, our findings suggest that a high proportion of Sudanese adults remain susceptible to VZV infection. This agrees with a previous study reporting a seroprevalence of 64% among 2196 adult Sudanese asylum seekers in Germany. In that study, Sudanese had the lowest VZV seroprevalence among all investigated nationalities and among individuals aged <45 years [7]. Our findings also agree with reports from other tropical countries, where varicella is acquired at a higher mean age [8], possibly because of the heat-labile nature of VZV that may influence exposure [1]. The nearly 21% susceptible HCWs identified in this study pose a risk to vulnerable patients such as severely immunocompromised people, pregnant women and premature infants [8]. In addition, more than 25% seronegative women of childbearing age are susceptible to primary infection during pregnancy with the risk of severe consequences for the child [1, 2]. Our findings suggest that both women of reproductive age and HCWs are important target groups for VZV immunity evaluation and vaccination [9], even though the two sequential vaccinations required pose an additional financial burden.

Although the samples used for this study had been collected in 2015 and 2016, there is no reason to believe that the sero-profiles from later time points look different, since there has been no change in varicella surveillance or prevention policy in Sudan since sample collection took place. Because data on varicella exposure are largely lacking for Africa, our findings on disease epidemiology are not only of local interest, but also to public health authorities and HCWs from similar settings without vaccination and case notification.

A limitation of our study to be addressed by future research is that the results are not necessarily representative of the whole Sudanese population since samples were collected from a limited geographical area and defined population groups, due to logistic and financial reasons. Although we cannot completely exclude cross-reactivity with antibodies against herpes simplex virus [10], the manufacturer reported no false positives among sera from 12 herpes simplex virus type 1 patients and an overall assay specificity of 100% in the ELISA kit manual.

In conclusion, the results of this first VZV study in Sudan suggest active virus circulation in different age groups. Especially HCWs at the start of their career would benefit from vaccination, not only to save themselves from herpes zoster and its sequelae, but also to indirectly protect vulnerable patients. Although data from Africa are largely lacking, the high susceptibility rate among women of childbearing age highlights the danger of varicella infection during pregnancy with potential sequelae in infants.

## Data Availability

The datasets used in the current study are available upon request from the corresponding author.

## References

[1] Gershon AA (2015) Varicella zoster virus infection. Nature Reviews Disease Primers 1, 15016. 10.1038/nrdp.2015.16 PMC5381807 27188665

[2] Heininger U and Seward JF (2006) Varicella. Lancet 368, 1365–1376. 17046469 10.1016/S0140-6736(06)69561-5

[3] Kennedy PGE and Gershon AA (2018) Clinical features of varicella-zoster virus infection. Viruses 10, 609. 30400213 10.3390/v10110609 PMC6266119

[4] World Health Organization (WHO) Database (2019). Available at https://www.who.int/docs/default-source/documents/immunization/data/vaccine-intro-status.pdf?sfvrsn=bb2857ec_2 (Accessed 15 May 2022).

[5] Adam O (2020) Seroprevalence of measles, mumps, and rubella and genetic characterization of mumps virus in Khartoum, Sudan. International Journal of Infectious Diseases 91, 87–93. 31759167 10.1016/j.ijid.2019.11.019

[6] Doshi RH (2018) Low varicella zoster virus seroprevalence among young children in the Democratic Republic of the Congo. Pediatric Infectious Disease Journal 37, 138–143. 28834954 10.1097/INF.0000000000001750 PMC5762406

[7] Toikkanen SE (2016) Seroprevalence of antibodies against measles, rubella and varicella among asylum seekers arriving in Lower Saxony, Germany, November 2014–October 2015. International Journal of Environmental Research and Public Health 13, 650. 27376309 10.3390/ijerph13070650 PMC4962191

[8] World Health Organization (2014) Varicella and herpes zoster vaccines: WHO position paper, June 2014. Weekly Epidemiological Record 89, 265–287. 24983077

[9] Shefer A (2011) Immunization of health-care personnel: recommendations of the Advisory Committee on Immunization Practices (ACIP). Morbidity and Mortality Weekly Report. Recommendations and Reports 60, 1–45. 22108587

[10] Bernstein DI (1990) Antibody response to herpes simplex virus glycoproteins gB and gD. Journal of Medical Virology 30, 45–49. 2154542 10.1002/jmv.1890300110

